# Hyperglycemia aggravates acute liver injury by promoting liver‐resident macrophage NLRP3 inflammasome activation via the inhibition of AMPK/mTOR‐mediated autophagy induction

**DOI:** 10.1111/imcb.12297

**Published:** 2019-11-19

**Authors:** Qi Wang, Song Wei, Shun Zhou, Jiannan Qiu, Chenyu Shi, Rui Liu, Haoming Zhou, Ling Lu

**Affiliations:** ^1^ Hepatobiliary Center The First Affiliated Hospital of Nanjing Medical University Research Unit of Liver Transplantation and Transplant Immunology Chinese Academy of Medical Sciences Nanjing China; ^2^ Key Laboratory of Liver Transplantation Chinese Academy of Medical Sciences Nanjing China; ^3^ NHC Key Laboratory of Living Donor Liver Transplantation Nanjing China; ^4^ School of Medical Southeast University Nanjing China

**Keywords:** AMPK, autophagy, hyperglycemia, Kupffer cell, liver injury, mTOR, NLRP3 inflammasome, thioacetamide

## Abstract

Although the detrimental effects of diabetes mellitus/hyperglycemia have been observed in many liver disease models, the function and mechanism of hyperglycemia regulating liver‐resident macrophages, Kupffer cells (KCs), in thioacetamide (TAA)‐induced liver injury remain largely unknown. In this study, we evaluated the role of hyperglycemia in regulating NOD‐like receptor family pyrin domain‐containing 3 protein (NLRP3) inflammasome activation by inhibiting autophagy induction in KCs in the TAA‐induced liver injury model. Type I diabetes/hyperglycemia was induced by streptozotocin treatment. Compared with the control group, hyperglycemic mice exhibited a significant increase in intrahepatic inflammation and liver injury. Enhanced NLRP3 inflammasome activation was detected in KCs from hyperglycemic mice, as shown by increased gene induction and protein levels of NLRP3, cleaved caspase‐1, apoptosis‐associated speck‐like protein containing a caspase recruitment domain and interleukin‐1β, compared with control mice. NLRP3 inhibition by its antagonist CY‐09 effectively suppressed inflammasome activation in KCs and attenuated liver injury in hyperglycemic mice. Furthermore, inhibited autophagy activation was revealed by transmission electron microscope detection, decreased LC3B protein expression and p‐62 protein degradation in KCs isolated from TAA‐stressed hyperglycemic mice. Interestingly, inhibited 5′ AMP‐activated protein kinase (AMPK) but enhanced mammalian target of rapamycin (mTOR) activation was found in KCs from TAA‐stressed hyperglycemic mice. AMPK activation by its agonist 5‐aminoimidazole‐4‐carboxamide ribonucleotide (AICAR) or mTOR signaling knockdown by small interfering RNA restored autophagy activation, and subsequently, inhibited NLRP3 inflammasome activation in KCs, leading to ultimately reduced TAA‐induced liver injury in the hyperglycemic mice. Our findings demonstrated that hyperglycemia aggravated TAA‐induced acute liver injury by promoting liver‐resident macrophage NLRP3 inflammasome activation via inhibiting AMPK/mTOR‐mediated autophagy. This study provided a novel target for prevention of toxin‐induced acute liver injury under hyperglycemia.

## Graphical abstract

We demonstrated that hyperglycemia aggravated acute liver injury induced by thioacetamide by promoting liver‐resident macrophage NOD‐like receptor family pyrin domain‐containing 3 protein inflammasome activation via inhibiting autophagy mediated by 5′ AMP activated protein kinase/mammalian target of rapamycin (AMPK/mTOR). This study provides a novel target for the prevention of toxin‐induced acute liver injury under hyperglycemia.

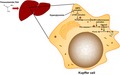

## Introduction

Diabetes mellitus is one of the most severe endocrine metabolic disorders that threatens human health worldwide.^1^ Hyperglycemia, the most prominent symptom of diabetes, has been shown to trigger acute and chronic inflammation.^2^, ^3^ Epidemiological studies have demonstrated that hyperglycemia is a major risk factor involved in liver injury.^4^, ^5^ Thioacetamide (TAA), a classic hepatotoxin, contributes to acute and chronic liver injury by inducing oxidative stress and sterile inflammation.^6^, ^7^ Studies have shown that hyperglycemia plays a critical role in liver injury.^4^, ^5^ However, whether hyperglycemia aggravates TAA‐induced acute liver injury remains unclear.

Liver‐resident macrophages, Kupffer cells (KCs), play an important role in the pathogenesis of toxin‐induced liver injury by mediating sterile liver inflammation.^8^, ^9^ KCs are guards that maintain liver homeostasis.^10^ During hepatic inflammation, KCs are activated and release chemokines and inflammatory cytokines. Then, KCs recruit other immune cells to the damaged tissue site and amplify the inflammatory signal.^11^

Several factors, including insulin resistance,^12^ oxidative stress,^13^ and endoplasmic reticulum stress,^5^ may explain the mechanisms related to pathological and functional changes in liver injury‐induced hyperglycemia. Metabolic disturbances during acute liver injury as a result of hyperglycemia exacerbate inflammatory responses, such as NOD‐like receptor family pyrin domain‐containing 3 protein (NLRP3) inflammasome activation.^14^ The NLRP3 inflammasome is a complex consisting of the NOD‐like receptor NLRP3, the adaptor molecule apoptosis‐associated speck‐like protein containing a caspase recruitment domain (ASC) and the enzyme caspase‐1.^15^, ^16^, ^17^, ^18^, ^19^ The activated NLRP3 inflammasome generates numerous inflammatory cytokines, such as interleukin (IL)‐1β and IL‐18,^20^, ^21^ which play a critical role in various chronic diseases, including diabetes.^22^

Recent studies showed that the inflammasome was regulated by autophagy,^23^ and autophagy suppressed intracellular signaling by clearing dysfunctional mitochondria that would otherwise increase the generation of reactive oxygen species.^24^ Excess reactive oxygen species can result in cellular inflammation and dysfunction. A study indicated that the inhibition of autophagy increased the production of IL‐1β in ARPE‐19 cells by reactive oxygen species‐induced NLRP3 inflammasome activation in hyperglycemia.^25^ Another study indicated that hyperglycemia inhibited Schwann cell autophagy and aggravates diabetic peripheral neuropathy.^26^ Whether hyperglycemia promotes the activation of the NLRP3 inflammasome by inhibiting autophagy in TAA‐induced acute liver injury remains unclear.

In this study, we determined that hyperglycemia promoted acute liver injury and inflammatory immune responses by promoting liver‐resident macrophage NLRP3 inflammasome activation via the inhibition of the 5′ AMP‐activated protein kinase/mammalian target of rapamycin (AMPK/mTOR) autophagy signaling pathways in a streptozotocin (STZ)‐induced hyperglycemic mouse model.

## Results

### Hyperglycemia exacerbates TAA‐induced acute liver injury

**Figure 1:**
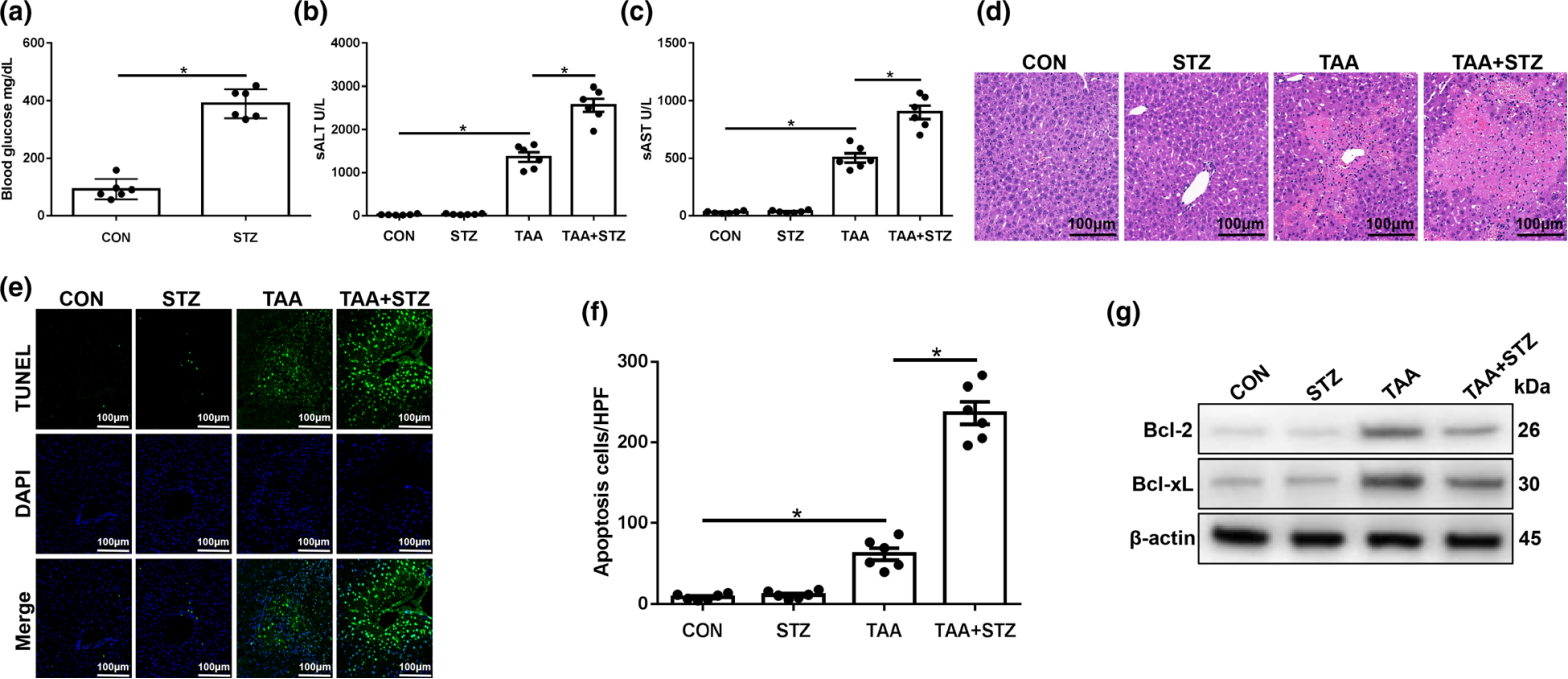
Hyperglycemia exacerbates thioacetamide (TAA)‐induced acute liver injury. Diabetic streptozotocin (STZ) and control (CON) mice were prepared as described in the “Methods” section. TAA‐induced acute liver injury or a sham procedure was performed. **(a)** Blood glucose levels were measured in different groups (*n* = 6 mice/group). **(b**–**d)** Serum alanine aminotransferase (sALT) and aspartate aminotransferase (sAST) levels (*n* = 6 mice/group) and liver histopathology (representative of six experiments) were used to evaluate the liver injury at 24 h after TAA administration. **(e)** Terminal deoxynucleotidyl transferase dUTP nick end labeling (TUNEL) staining of the liver sections (200× magnification, representative of six mice/group). **(f)** The relative ratio of TUNEL‐positive cells in different groups (*n* = 6 mice/group). **(g)** The levels of Bcl‐2, Bcl‐xL and β‐actin proteins were measured by western blot. Representative of three experiments. *P < 0.05. DAPI, 4′,6‐diamidino‐2‐phenylindole.

To examine the role of diabetes/hyperglycemia in the development of TAA‐induced acute liver injury, we induced hyperglycemia in C57BL/6J mice with repetitive injections of low‐dose STZ. Hyperglycemia was confirmed in these treated mice on day 14 postinjection with STZ (Figure 1a). Compared with TAA treatment alone, STZ pretreatment aggravated liver injury as evidenced by increased serum alanine aminotransferase (ALT) and aspartate transaminase (AST) levels (Figure 1b, c), as well as aggravated TAA‐induced acute liver injury (Figure 1d) and augmented hepatocellular apoptosis (Figure 1e, f). Significantly lower levels of antiapoptotic proteins, such as Bcl‐2 and Bcl‐xL, were also observed in livers from TAA + STZ‐treated mice compared with TAA‐only treated mice (Figure 1g). These results demonstrated that hyperglycemia aggravated TAA‐induced acute liver injury.

### Hyperglycemia aggravates TAA‐induced acute liver injury by increasing NLRP3 inflammasome activation in KCs

**Figure 2:**
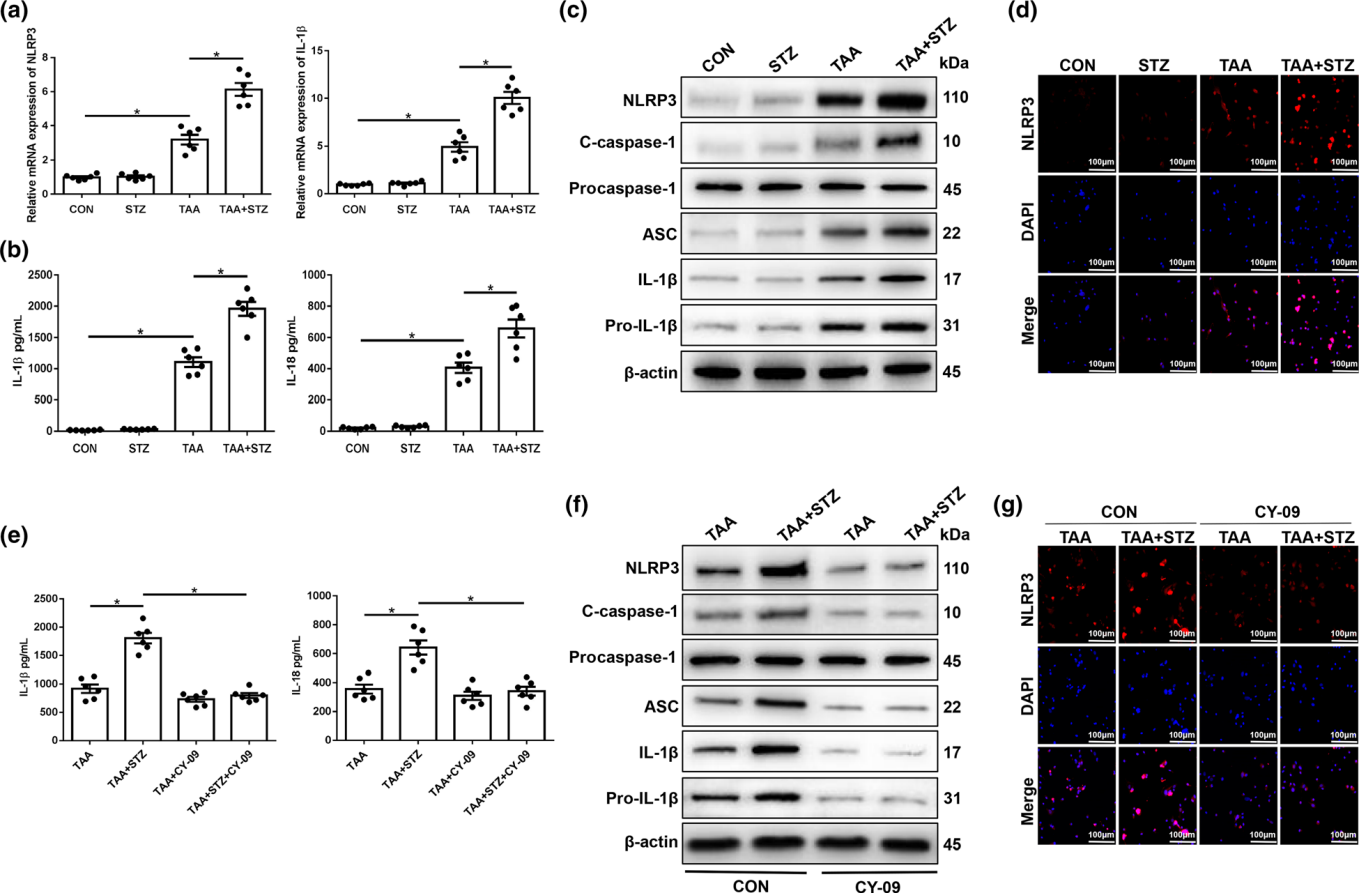
Hyperglycemia induces the expression of the NLRP3 inflammasome in Kupffer cells (KCs) in thioacetamide (TAA)‐induced acute liver injury mice. Mice were subjected to streptozotocin (STZ) pretreatment and TAA administration as described in the “Methods” section. KCs were isolated from different experimental groups. **(a)** The expression of proinflammatory genes in KCs was measured by quantitative real‐time‐PCR (*n* = 6 mice/group). **(b)** Isolated KCs from different experimental groups were cultured for 6 h. Interleukin‐1 β (IL‐1β) and IL‐18 protein levels were measured in the culture supernatant by ELISA (*n* = 6 mice/group). **(c)** The levels of intracellular NLRP3, cleaved caspase‐1, procaspase‐1, ASC, IL‐1β, pro‐IL‐1β and β‐actin proteins were measured by western blot (representative of three experiments). **(d)** Immunofluorescence staining of NLRP3 in KCs (200× magnification, representative of three experiments). Diabetic and control mice were treated with the NLRP3 inhibitor CY‐09 (20 mg kg^−1^, intraperitoneally) once a day for 7 days prior to TAA administration. **(e)** Isolated KCs from different experimental groups were cultured for 6 h. IL‐1β and IL‐18 protein levels were measured in the culture supernatant by ELISA (*n* = 6 mice/group). **(f)** The levels of intracellular NLRP3, cleaved caspase‐1, procaspase‐1, ASC, IL‐1β, pro‐IL‐1β and β‐actin proteins were detected by western blot (representative of three experiments). **(g)** Immunofluorescence staining of NLRP3 in KCs (200× magnification, representative of three experiments). *P < 0.05. CON, control; DAPI, 4′,6‐diamidino‐2‐phenylindole; mRNA, messenger RNA; NLRP3, NOD‐like receptor family pyrin domain‐containing 3 protein.

To evaluate the role of hyperglycemia in affecting the inflammatory response of KCs in TAA‐induced liver injury, we compared NLRP3 inflammasome activation in KCs isolated from different groups. Interestingly, significantly increased mRNA levels of NLRP3 and IL‐1β, culture supernatant levels of IL‐1β and IL‐18 and protein levels of NLRP3, cleaved caspase‐1, ASC, IL‐1β and pro‐IL‐1β were found in KCs isolated from hyperglycemic mice after 24 h of TAA treatment (Figure 2a–c). The KCs isolated from TAA + STZ mice also showed increased NLRP3 immunofluorescence staining (Figure 2d). Thus, hyperglycemia induced NLRP3 inflammasome activation in liver KCs post‐TAA treatment.

**Figure 3:**
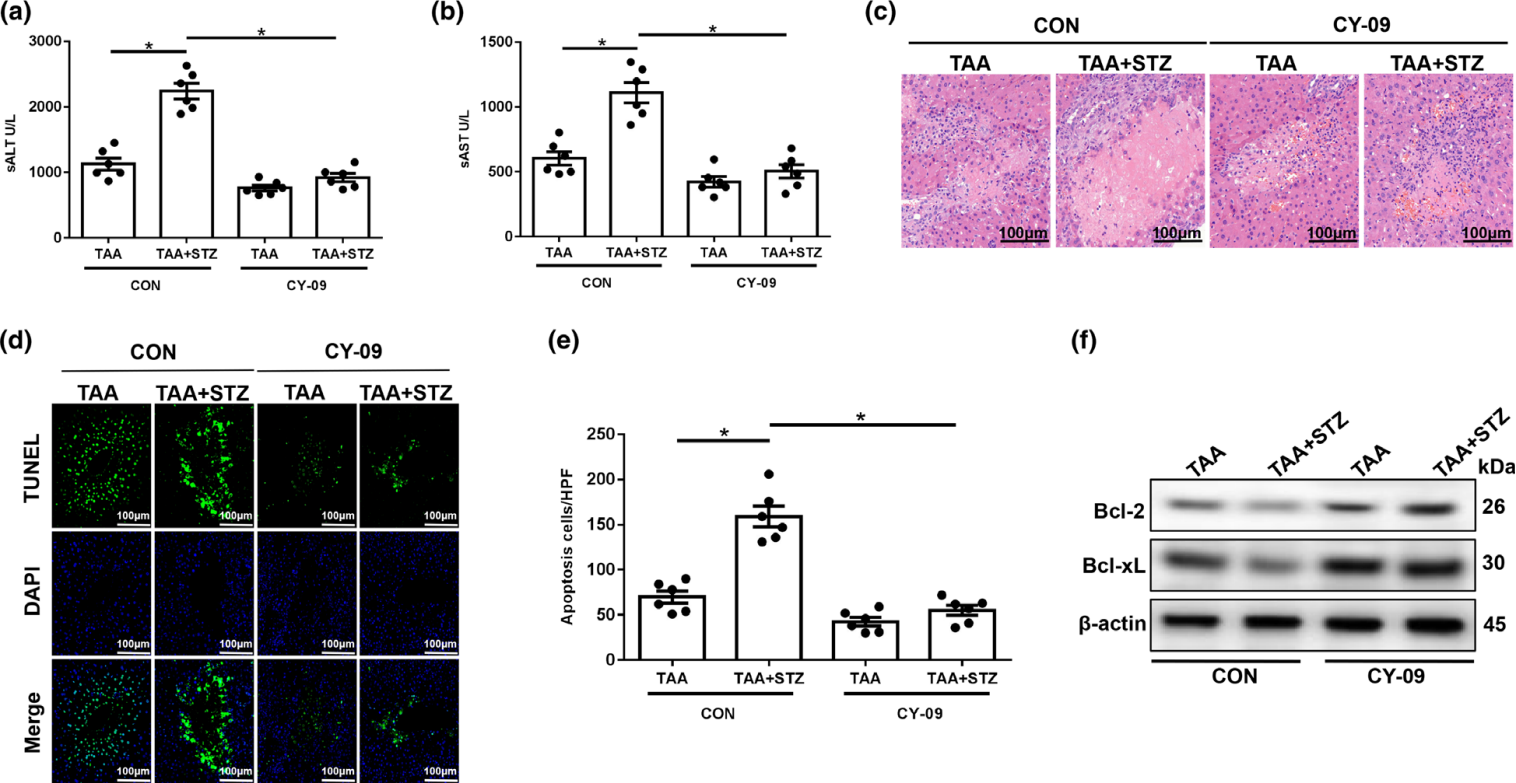
NLRP3 inhibitor treatment alleviates thioacetamide (TAA)‐induced acute liver injury in hyperglycemic mice. **(a–c)** Serum alanine aminotransferase (sALT), aspartate aminotransferase (sAST) levels (*n* = 6 mice/group) and liver histopathology (representative of six experiments) were used to evaluate liver injury in diabetic mice and controls after treatment with CY‐09 and TAA. **(d)** Terminal deoxynucleotidyl transferase dUTP nick end labeling (TUNEL) staining of liver sections (200× magnification, representative of six experiments). **(e)** The ratio of TUNEL‐positive cells in different experimental groups (*n* = 6 mice/group). **(f)** The levels of Bcl‐2, Bcl‐xL and β‐actin proteins were measured by western blot (representative of three experiments). *P < 0.05. CON, control; DAPI, 4′,6‐diamidino‐2‐phenylindole; HPF, high‐power field; NLRP3, NOD‐like receptor family pyrin domain‐containing 3 protein; STZ, streptozotocin.

To study the functional role of enhanced NLRP3 inflammasome activation in hyperglycemic KCs during TAA‐induced liver injury, CY‐09 was used to block NLRP3 inflammasome activation. A study showed that CY‐09 directly bound to the ATP‐binding site of the NLRP3 NACHT domain and inhibited its ATPase, oligomerization and activation. CY‐09 specifically blocks NLRP3 inflammasome activation in macrophages and had remarkable therapeutic effects on mouse models of NLRP3‐driven disease.^27^ Indeed, CY‐09 pretreatment effectively inhibited NLRP3 inflammasome activation in KCs from the TAA + STZ group, as evidenced by significantly decreased culture supernatant levels of IL‐1β and IL‐18 (Figure 2e) and protein expression levels of NLRP3, ASC, cleaved caspase‐1, IL‐1β and pro‐IL‐1β (Figure 2f), as well as decreased NLRP3 fluorescence intensity (Figure 2g). More importantly, NLRP3 inhibition protected against TAA‐induced liver injury, as shown by decreased serum levels of ALT (Figure 3a) and AST (Figure 3b), reduced liver architecture damage (Figure 3c) and a reduced number of terminal deoxynucleotidyl transferase dUTP nick end labeling (TUNEL)‐positive hepatocytes (Figure 3d, e). CY‐09 pretreatment also increased the protein levels of the antiapoptotic proteins Bcl‐2 and Bcl‐xL in livers from mice in the TAA + STZ group (Figure 3f). These results suggested that hyperglycemia aggravated TAA‐induced acute liver injury by inducing NLRP3 inflammasome activation in KCs.

### Hyperglycemia induces NLRP3 inflammasome activation by inhibiting mTOR‐mediated KC autophagy in TAA‐induced acute liver injury

**Figure 4:**
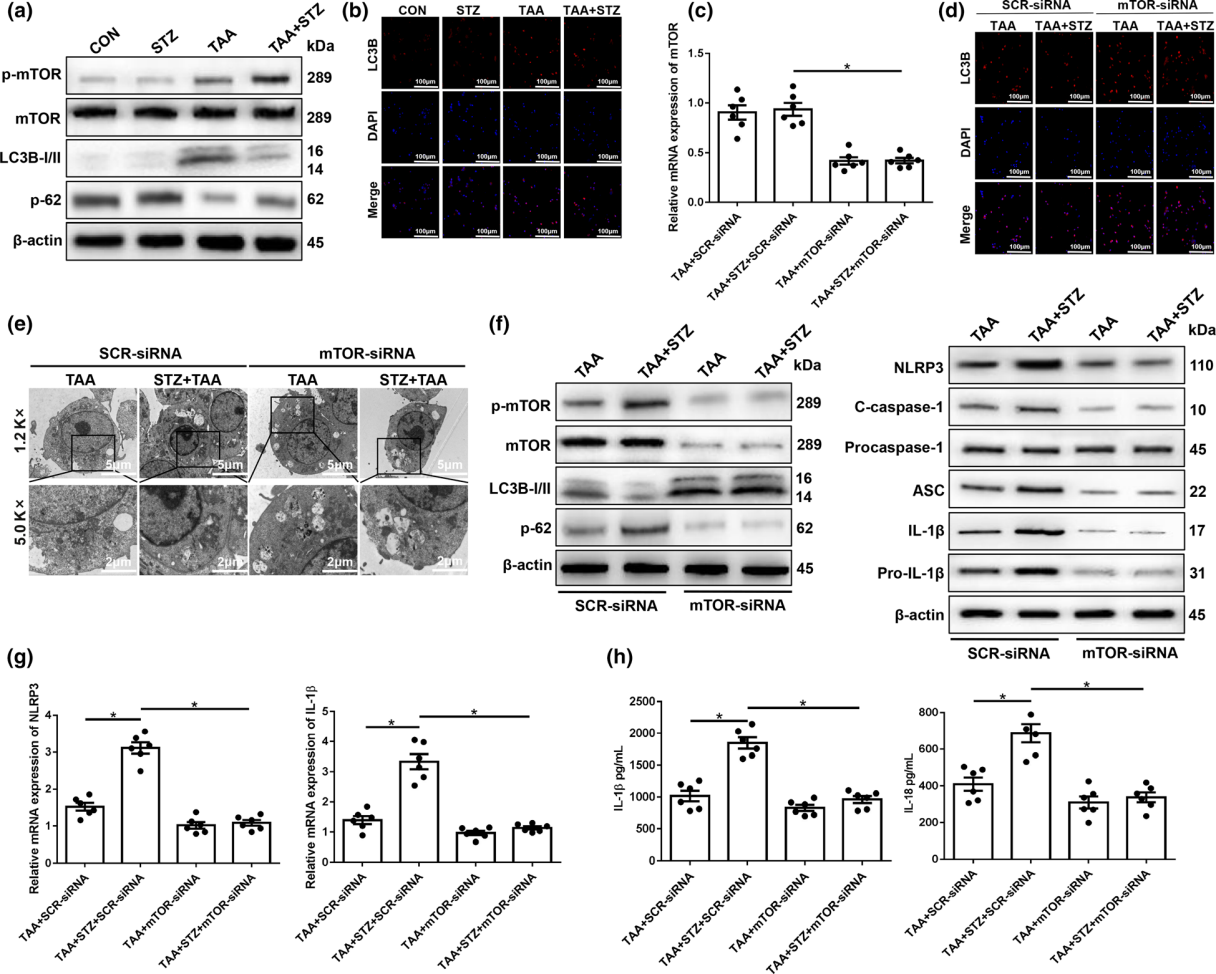
Hyperglycemia inhibits mammalian target of rapamycin (mTOR)‐mediated autophagy in Kupffer cells (KCs) post‐ thioacetamide (TAA) treatment. **(a)** After 24 h of TAA treatment, KCs were isolated, and intracellular p‐mTOR, mTOR, LC3B, p62 and β‐actin protein levels were detected by western blot (representative of three experiments). **(b)** The expression of LC3B in KCs was measured by immunofluorescence staining (200× magnification, representative of three experiments). Diabetic and control mice were injected with mannose‐conjugated mTOR‐siRNA (2 mg kg^−1^) via the tail vein at 4 h prior to TAA treatment. **(c)** The knockdown efficiency of mannose‐conjugated mTOR‐siRNA was measured by PCR (*n* = 6 mice/group). **(d)** Immunofluorescence staining of LC3B in KCs from different experimental groups (200× magnification, representative of three experiments). **(e)** Autophagic microstructures in cells were detected by transmission electron microscopy ( images are representative of those from three experiments). The areas enclosed within black squares were further amplified (1200× and 5000× magnification; scale bars, 5 and 2 μm). **(f)** The levels of intracellular p‐mTOR, mTOR, LC3B, p62, NLRP3, cleaved caspase‐1, procaspase‐1, ASC, interleukin‐1β (IL‐1β), pro‐IL‐1β and β‐actin proteins were detected by western blot (representative of three experiments). **(g)** The expression of proinflammatory genes in KCs was measured by quantitative real‐time‐PCR (*n* = 6 mice/group). **(h)** Isolated KCs from different experimental groups were cultured for 6 h. IL‐1β and IL‐18 protein levels were measured in the culture supernatant by ELISA (*n* = 6 mice/group). *P < 0.05. CON, control; DAPI, 4′,6‐diamidino‐2‐phenylindole; mRNA, messenger RNA; siRNA, small interfering RNA; STZ, streptozotocin.

Growing evidence indicates that autophagy plays a role in regulating NLRP3 inflammasome activation in immune cells. Thus, we further analyzed autophagy activation in KCs from different groups. As shown in Figure 4a, KCs isolated from the TAA + STZ group showed higher protein levels of p‐mTOR than those from the TAA group. LC3B and p62 are representative autophagy markers. Western blot results showed that in KCs isolated from the TAA + STZ group, LC3B expression was lower and, p62 expression was higher than that in KCs from the TAA group (Figure 4a). Decreased LC3B intensity in the immunofluorescence staining similarly indicated that hyperglycemia inhibited KC autophagy post‐TAA treatment (Figure 4b).

To further examine the role of mTOR in regulating hyperglycemia‐induced KC autophagy activation, mannose‐conjugated polymers were employed to deliver mTOR small interfering RNA (siRNA) or scrambled control siRNA *in vivo*. Mannose‐mediated siRNA is specifically delivered to macrophages/KCs because they express a mannose‐specific membrane receptor.^28^ The knockdown efficiency of mannose‐conjugated mTOR siRNA is shown in Figure 4c. Indeed, mTOR siRNA effectively inhibited mTOR activation and increased autophagy in KCs isolated from TAA‐induced hyperglycemic mice, as indicated by the increased fluorescence intensity of LC3B staining (Figure 4d). To further confirm the inhibition of autophagy by hyperglycemia in KCs, transmission electron microscopy was used to detect the characteristic autophagosomes with double‐membrane structures or autolysosomes generated by the fusion of autophagosomes with lysosomes. Indeed, mTOR knockdown improved cellular autophagosome (AP) and autolysosome (AL) formation in KCs from the TAA + STZ group (Figure 4e). Western blot analysis of LC3B and p62 further confirmed the restoration of autophagy by mTOR knockdown in KCs from the TAA + STZ group (Figure 4f). Furthermore, mTOR knockdown inhibited NLRP3 inflammasome activation in KCs isolated from TAA‐exposed hyperglycemic mice, as evidenced by significantly decreased protein expression levels of NLRP3, cleaved caspase‐1, ASC, IL‐1β and pro‐IL‐1β (Figure 4f), mRNA levels of NLRP3 and IL‐1β (Figure 4g) and culture supernatant levels of IL‐1β and IL‐18 (Figure 4h).

**Figure 5:**
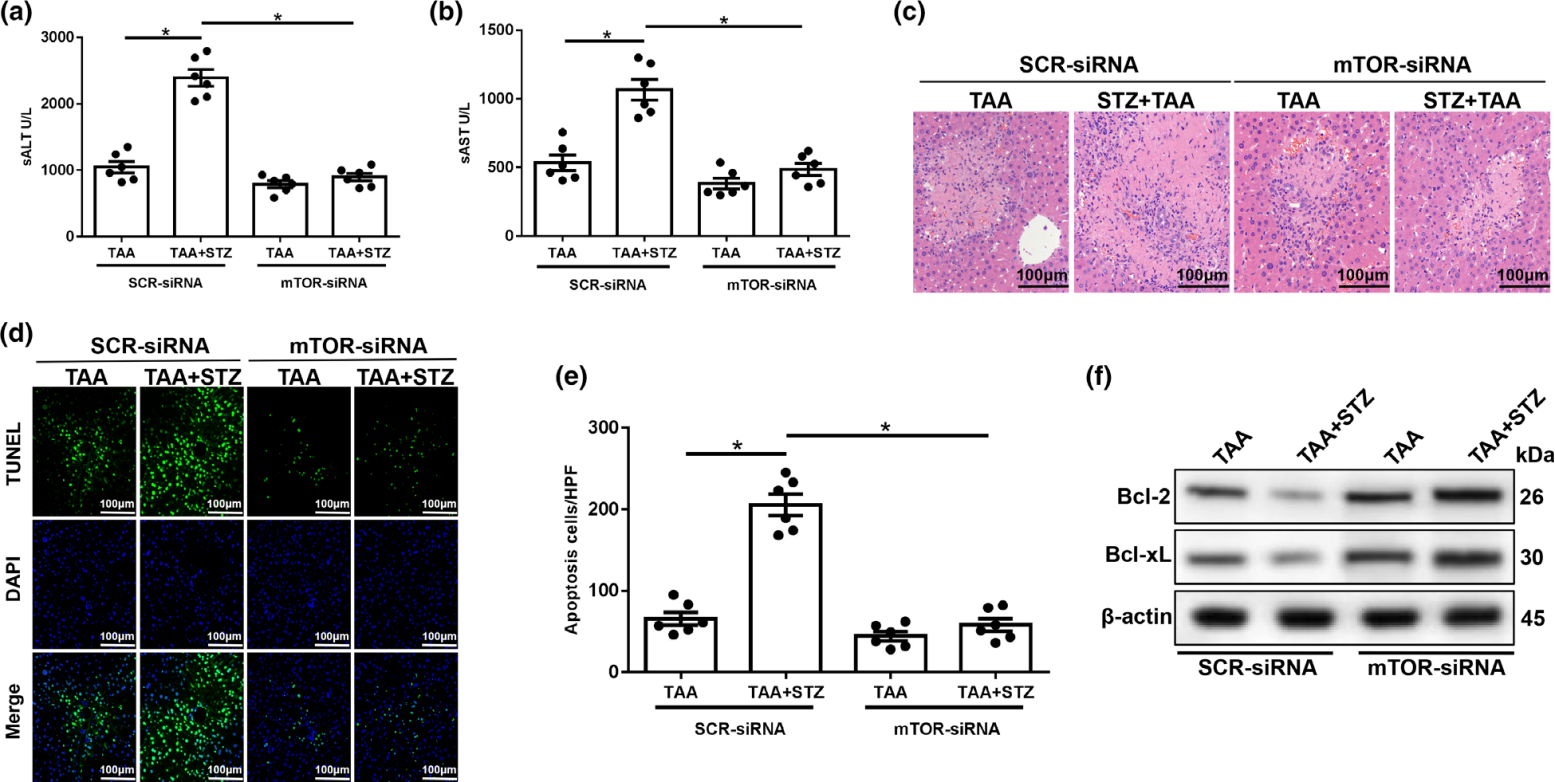
Mammalian target of rapamycin (mTOR) knockdown in Kupffer cells (KCs) alleviates thioacetamide (TAA)‐induced acute liver injury. **(a–c)** Serum alanine aminotransferase (sALT), aspartate aminotransferase (sAST) levels (*n* = 6 mice/group) and liver histopathology (representative of six experiments) were used to evaluate liver injury in diabetic mice and controls after treatment with mTOR‐siRNA and TAA. **(d)** Terminal deoxynucleotidyl transferase dUTP nick end labeling (TUNEL) staining of liver sections (200× magnification, representative of six experiments). **(e)** The ratio of TUNEL‐positive cells in different experimental groups (*n* = 6 mice/group). **(f)** The levels of Bcl‐2, Bcl‐xL and β‐actin proteins were measured by western blot (representative of three experiments). *P < 0.05. HPF, high‐power field; sALT, serum alanine aminotransferase; sAST, aspartate aminotransferase; siRNA, small interfering RNA; STZ, streptozotocin.

More importantly, mTOR knockdown protected against TAA‐induced liver injury, as shown by decreased serum levels of ALT (Figure 5a) and AST (Figure 5b), reduced damage to the liver architecture (Figure 5c) and a reduced number of TUNEL‐positive hepatocytes (Figure 5d, e). mTOR knockdown also increased the levels of the antiapoptotic proteins Bcl‐2 and Bcl‐xL in livers after TAA pretreatment (Figure 5f). Our results demonstrated that hyperglycemia induced NLRP3 inflammasome activation by inhibiting mTOR‐mediated autophagy in KCs in TAA‐induced acute liver injury.

### AMPK/mTOR‐mediated autophagy inhibition promotes NLRP3 inflammasome activation in KCs during TAA‐induced acute liver injury in hyperglycemic mice

**Figure 6:**
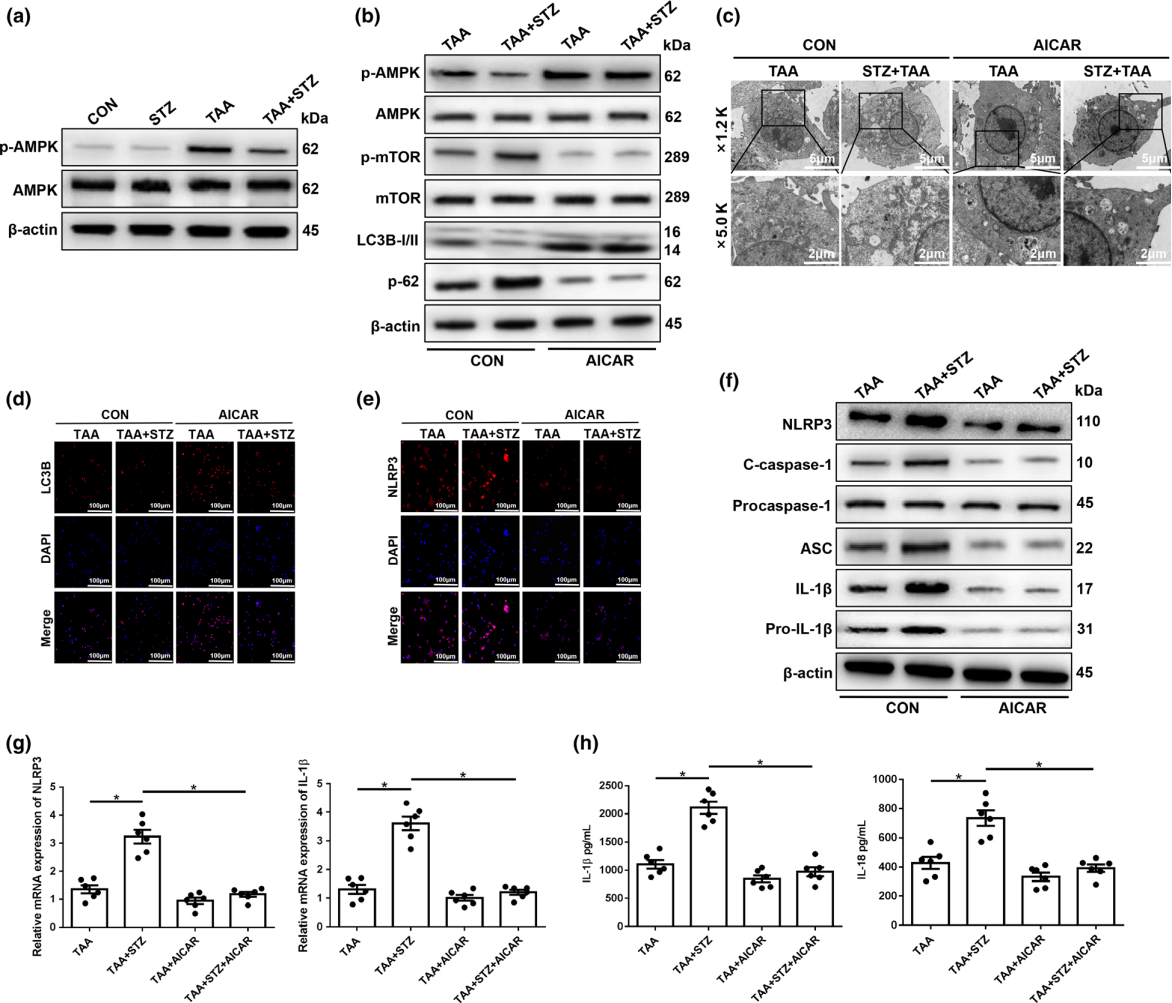
The inhibition of 5′ AMP‐activated protein kinase (AMPK) under hyperglycemic conditions suppresses mammalian target of rapamycin (mTOR)‐dependent autophagy and promotes the expression of the NLRP3 inflammasome in Kupffer cells (KCs). **(a)** The levels of intracellular p‐AMPK and β‐actin proteins were measured by western blot (representative of three experiments). Diabetic mice and controls were subjected to AMPK activator (AICAR, 100 mg kg^−1^, intraperitoneally) treatment once a day for 7 days prior to thioacetamide (TAA) administration. **(b)** The levels of intracellular p‐AMPK, AMPK, p‐mTOR, mTOR, LC3B, p62 and β‐actin proteins were detected by western blot (representative of three experiments). **(c)** The detection of autophagic microstructures in KCs by transmission electron microscopy; the areas enclosed within black squares were further amplified (1200× and 5000× magnification; scale bars, 5 and 2 μm; representative of three experiments). **(d** and **e)** Immunofluorescence staining of NLRP3 and LC3B in KCs (200× magnification; representative of three experiments). **(f)** The levels of intracellular NLRP3, cleaved caspase‐1, procaspase‐1, ASC, interleukin‐1β (IL‐1β), pro‐IL‐1β and β‐actin proteins were measured by western blot (representative of three experiments). **(g)** The expression of proinflammatory genes in KCs was detected by quantitative real‐time‐PCR (*n* = 6 mice/group). **(h)** Isolated KCs from different experimental groups were cultured for 6 h. IL‐1β and IL‐18 protein levels were measured in the culture supernatant by ELISA (*n* = 6 mice/group). *P < 0.05. AICAR, 5‐aminoimidazole‐4‐carboxamide ribonucleotide; CON, control; DAPI, 4′,6‐diamidino‐2‐phenylindole; mRNA, messenger RNA; NLRP3, NOD‐like receptor family pyrin domain‐containing 3 protein; STZ, streptozotocin.

The essential role of AMPK signaling in regulating autophagy and acute liver injury has recently been reported.^29^ Thus, we first tested the activation of AMPK in hyperglycemic KCs from TAA‐exposed livers and found that the protein level of p‐AMPK was significantly lower in the TAA + STZ group than the TAA group (Figure 6a).

To explore the functional role of AMPK in regulating mTOR, thereby affecting autophagy activation in hyperglycemic KCs, the AMPK agonist 5‐aminoimidazole‐4‐carboxamide ribonucleotide (AICAR) was utilized to restore AMPK activity. As shown in Figure 6b, pretreatment with AICAR effectively increased AMPK activation and decreased mTOR activation in KCs from both normoglycemic and hyperglycemic mice post‐TAA exposure, as evidenced by the protein levels of p‐AMPK and p‐mTOR (Figure 6b). Notably, AMPK activation significantly restored the activation of autophagy in KCs isolated from hyperglycemic mice after TAA exposure, as evidenced by increased LC3B but decreased p62 protein levels (Figure 6b), which was further confirmed by transmission electron microscopy analysis and LC3B immunofluorescence staining (Figure 6c, d). Furthermore, the inhibition of NLRP3 inflammasome activation by AICAR pretreatment was detected in KCs isolated from hyperglycemic mice after TAA exposure, as evidenced by the decreased immunofluorescence intensity of NLRP3 staining (Figure 6e), as well as the protein expression levels of NLRP3, cleaved caspase‐1, ASC, IL‐1β and pro‐IL‐1β (Figure 6f), gene expression levels of NLRP3 and IL‐1β (Figure 6g) and the culture supernatant levels of IL‐1β and IL‐18 (Figure 6h).

**Figure 7:**
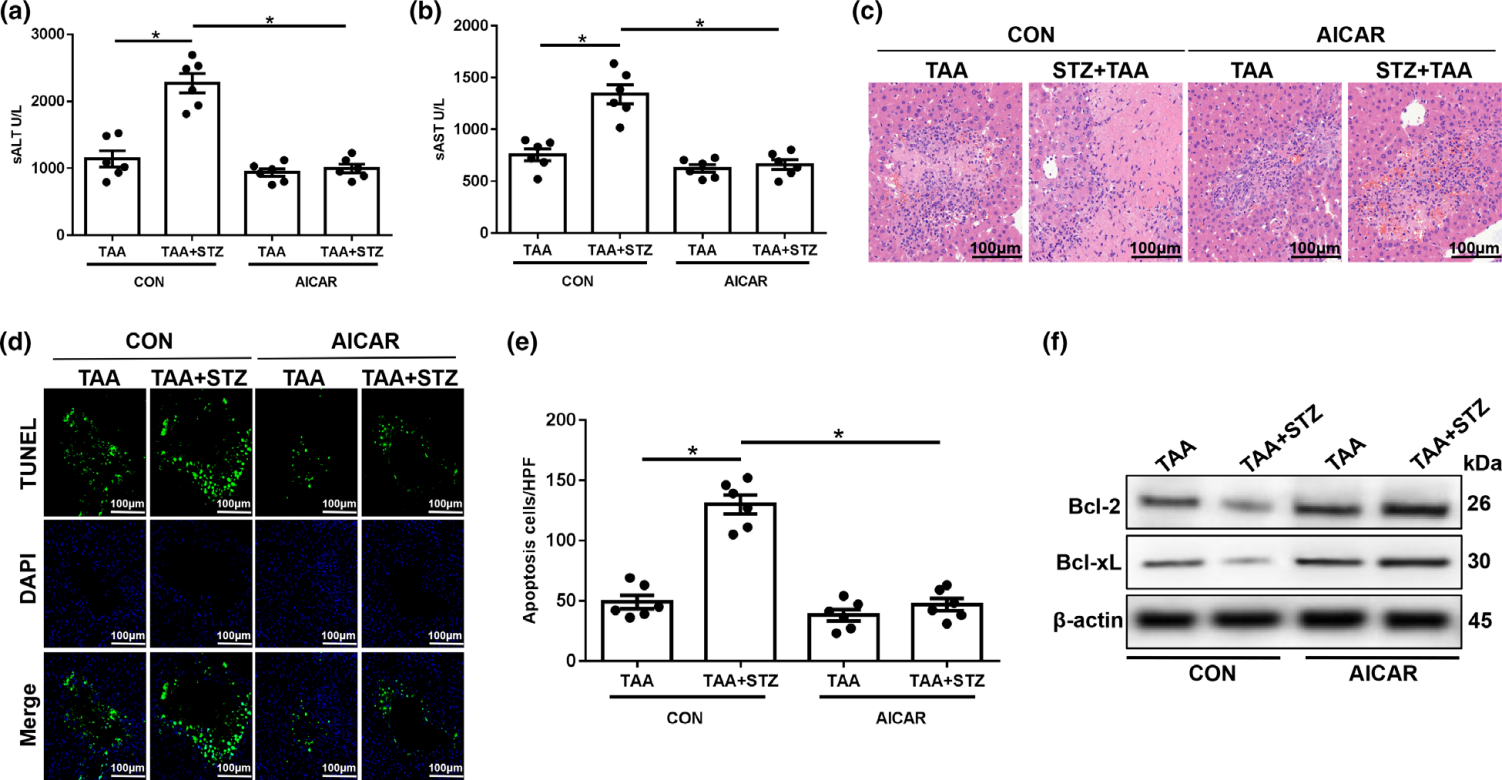
5′ AMP‐activated protein kinase (AMPK) activator (AICAR) treatment alleviates thioacetamide (TAA)‐induced acute liver injury in hyperglycemic mice. **(a–c)** Serum levels of aspartate transaminase (sAST) and alanine aminotransferase (sALT; *n* = 6 mice/group) and liver histopathology (representative of six experiments) were used to evaluate liver injury in diabetic mice and controls post‐AICAR and TAA treatment. **(d)** Terminal deoxynucleotidyl transferase dUTP nick end labeling (TUNEL) staining of liver sections (200× magnification, representative of six experiments). **(e)** The relative ratio of TUNEL‐positive cells in different groups (*n* = 6 mice/group). **(f)** The levels of Bcl‐2, Bcl‐xL and β‐actin proteins were measured by western blot (representative of three experiments). *P < 0.05. AICAR, 5‐aminoimidazole‐4‐carboxamide ribonucleotide; CON, control; NLRP3, NOD‐like receptor family pyrin domain‐containing 3 protein; STZ, streptozotocin.

Finally, we evaluated the effects of autophagy restoration by AMPK activation on TAA‐induced liver injury. AICAR pretreatment significantly alleviated liver injury, as indicated by decreased serum levels of ALT and AST, and reduced liver architecture damage and hepatocellular cell death (Figure 7a–e, TAA + STZ + AICAR *versus* TAA + STZ). Significantly increased levels of the antiapoptotic proteins Bcl‐2 and Bcl‐xL were also observed in TAA + STZ + AICAR livers compared with TAA + STZ livers (Figure 7f). By contrast, no notable protection by AICAR pretreatment was found in normoglycemic control mice (Figure 7a–f, TAA + AICAR *versus* TAA). In conclusion, these results showed that hyperglycemia induced NLRP3 inflammasome activation by inhibiting AMPK/mTOR‐mediated autophagy activation in KCs in TAA‐induced acute liver injury (Supplementary figure 1).

## Discussion

Diabetes and the accompanying hyperglycemia have been shown to exacerbate acute tissue injuries in the hearts,^30^ lungs,^31^ kidneys^32^ and nerves.^33^ Hyperglycemia has been shown to aggravate acute liver injury in many models. For example, a study showed that hyperglycemia aggravated hepatic warm ischemia and reperfusion injury by inducing hyperinflammatory immune responses via the activation of the AGE‐RAGE signaling pathway in KCs.^34^ Another study showed that hyperglycemia aggravated hepatic warm ischemia and reperfusion injury by inhibiting liver‐resident macrophage M2 polarization via C/EBP homologous protein‐mediated endoplasmic reticulum stress.^5^ However, the effects of hyperglycemia on TAA‐induced liver injury remain largely unknown. We address the question of whether and how hyperglycemia impacts TAA‐induced acute liver injury, focusing on its effects on the proinflammatory activation of KCs. To the best of our knowledge, this is the first study to demonstrate that hyperglycemia aggravates TAA‐induced acute liver injury by promoting liver‐resident macrophage NLRP3 inflammasome activation via the inhibition of AMPK/mTOR‐mediated autophagy induction.

TAA is a thiono‐sulfur‐containing compound that has liver‐damaging and carcinogenic effects. Studies showed that the treatment of rats with TAA caused oxidative stress and reactive oxygen species‐induced NF‐κB activation, which mediated liver damage and inflammation.^35^, ^36^ Another study showed that TAA exacerbated liver injury by inducing the phosphorylation of the procarcinogenic proteins phosphoinositide 3‐kinase, protein kinase B (Akt) and mTOR in hepatocytes.^37^ Other studies have shown that chronic TAA administration could induce liver fibrosis, activate hepatic stellate cells,^38^ downregulate CD4^+^ or CD8^+^ T‐cell expansion and induce Foxp3^+^ regulatory T‐cell production.^39^ In addition, studies showed that ischemia and reperfusion injury could aggravate liver inflammation^28^ and that hyperglycemia suppressed KC M2 polarization and aggravated hepatic ischemia and reperfusion injury.^5^ However, whether hyperglycemia exacerbates TAA‐induced acute liver injury remains unclear.

KCs, which reside within the liver sinusoid, serve as guards for liver homeostasis; they produce inflammatory cytokines and various biological molecules.^40^ KCs play a critical role in lipopolysaccharide‐mediated liver inflammation by inducing hepatic granuloma formation and increasing tissue factor expression.^40^ Previous studies also reported that KCs negatively regulated acute liver injury induced by acetaminophen. The liver injury in KC‐ablated mice was more severe than that in KC‐sufficient mice.^41^, ^42^, ^43^ We previously showed that TAA‐induced acute liver injury promoted the proinflammatory response of KCs.^44^ However, whether hyperglycemia exacerbates TAA‐induced acute liver injury by mediating the KC inflammatory response remains unclear.

The NLRP3 inflammasome is a member of the inflammasome family, and includes the NOD‐like receptor NLRP3, the adaptor ASC and the effector enzyme caspase‐1.^45^, ^46^, ^47^ A novel study demonstrated that NLRP3 in KCs played a critical role in nonalcoholic steatohepatitis development by increasing proinflammatory cytokine IL‐1β secretion induced by palmitic acid stimulation.^48^ Another study suggested that NLRP3 inflammasome assembly inhibition through the NAD^+^/SIRT2 pathway attenuated nonalcoholic fatty liver disease in mice.^49^ However, the effect of hyperglycemia on NLRP3 activation in KCs remains unknown. In the present study, we found that hyperglycemia significantly increased NLRP3 inflammasome activation and inhibited KC autophagy in TAA‐treated mice. NLRP3 inhibition effectively suppressed the inflammasome activation in KCs and attenuated the liver injury in hyperglycemic mice.

Autophagy is a lysosomal degradation process that degrades cytoplasmic materials, such as misfolded proteins and dysfunctional organelles, in response to various stress conditions.^50^, ^51^ The role of macrophage autophagy in liver diseases remains poorly defined, but increasing evidence demonstrates that macrophage autophagy alleviates acute liver injury. The loss of autophagy‐related protein 4B in old mice aggravated liver ischemia and reperfusion injury.^52^ Macrophage autophagy limited acute toxin‐induced liver injury and death by suppressing the production of the proinflammatory cytokine IL‐1β.^53^ Hepatic autophagy deficiency compromised FXR functionality and caused cholestatic injury.^54^ We previously demonstrated that KC autophagy induction inhibited M1 polarization and promoted M2 polarization, leading to decreased TAA‐induced acute liver injury.^44^

Emerging evidence shows that autophagy can inhibit NLRP3 inflammasome activation. NLRP3 inflammasome activation was triggered by impaired mitophagy during the progression from nonalcoholic fatty liver disease to nonalcoholic steatohepatitis.^55^ The activation of autophagy by AMP‐activated protein kinase–TFEB (transcription factor EB) ameliorated steatohepatitis and NLRP3 inflammasome inhibition.^56^ The enhancement of macrophage autophagy by dietary polyunsaturated fatty acids inhibited NLRP3 inflammasome activation.^57^ Autophagy alleviated acute liver injury by inhibiting NLRP3 inflammasome activation in mice.^58^ Here, we found that autophagy activation was inhibited in KCs isolated from TAA‐treated hyperglycemic mice. The restoration of autophagy by AMPK activation or mTOR knockdown inhibited NLRP3 inflammasome activation in KCs and reduced the TAA‐induced liver injury in hyperglycemic mice.

In conclusion, the results of this study demonstrated that hyperglycemia inhibited AMPK/mTOR‐mediated autophagy induction, leading to enhanced NLRP3 inflammasome activation in KCs and increased TAA‐induced acute liver injury. Our findings provide a potential strategy for alleviating toxin‐induced acute liver injury in patients with diabetes/hyperglycemia.

## Methods

### Animals

Wild‐type C57BL/6J male mice (6 weeks old) were purchased from the Laboratory of Animal Resources of Nanjing Medical University and housed under specific pathogen‐free conditions with access to properly sterilized water and food. This study was performed in strict accordance with the recommendations in the protocol (number NMU08‐092), which was approved by the Institutional Animal Care and Use Committee of Nanjing Medical University.

### Mouse hyperglycemia and TAA‐induced acute liver injury model

Separate groups of mice were injected intraperitoneally with STZ (Sigma, St. Louis, MO, USA) at a dose of 40 mg kg^−1^ for 5 consecutive days or the same volume of vehicle control (sodium citrate buffer). Blood glucose levels from the tail vein were examined on day 14 (9 days after the last injection). Levels of blood glucose ≥300 mg dL^−1^ in mice were considered indicative of hyperglycemia. Mice were randomly separated into four groups: control group, STZ group, TAA group and TAA + STZ group (*n* = 8 mice/group). TAA (Sigma) dissolved in PBS was intraperitoneally injected into the mice at 300 mg kg^−1^. The same volume of PBS was intraperitoneally injected into the control mice. The mice were killed 24 h after TAA treatment. Blood samples and liver specimens were collected. Tissues were preserved in liquid nitrogen. Samples from the dissected livers were fixed in 10% neutral buffered formalin for further experiments.

### Hepatocellular function assay

Blood samples were centrifuged to obtain serum, and the levels of ALT and AST were detected by an automatic chemical analyzer (Olympus Company, Tokyo, Japan).

### TUNEL staining

Sections of paraffin‐embedded hepatic tissues were deparaffinized in toluene and then dehydrated in ethanol solutions. TUNEL staining of the liver samples was performed using a fluorescence detection kit (Roche, Basel, Switzerland) in accordance with the manufacturer's protocols.

### *In vivo* mTOR knockdown

mTOR siRNA (Santa Cruz, CA, USA) was mixed with mannose‐conjugated polymers (Polyplus‐transfection, Illkirch, France) according to the manufacturer's instructions and was injected via the tail vein (2 mg kg^−1^) 4 h prior to TAA administration.

### Histopathology and immunofluorescence staining

Liver samples were collected and stained with hematoxylin and eosin. Tissue damage and inflammation were observed by light microscopy. NLRP3 and LC3B in KCs were identified by immunofluorescence using an antirabbit NLRP3 mAb, an antirabbit LC3B mAb (Cell Signaling Technology, MA, USA) and a secondary goat antirabbit Texas Red‐conjugated IgG (Sigma) according to the manufacturer's instructions. 4′,6‐diamidino‐2‐phenylindole was used for nuclear staining. Positive cells were blindly observed in 10 high‐power fields/section (200×).

### Cell isolation and culture

KCs were isolated as previously described.^59^ In brief, livers were perfused *in situ* via the portal vein with warmed (37°C) Hanks’ balanced salt solution, followed by collagenase IV (Sigma). Perfused livers were dissected and teased through 70‐mm nylon mesh cell strainers (BD Biosciences, San Diego, CA, USA). Nonparenchymal cells were separated from hepatocytes by three 2‐min centrifugations at 50 *g*. Nonparenchymal cells were suspended in Hanks’ balanced salt solution and layered onto a 50%/25% two‐step Percoll gradient (Sigma) and centrifuged at 1800*g* at 4°C for 15 min. KCs in the middle layer were collected and allowed to attach to cell culture plates in Dulbecco's Modified Eagle Medium with 10% FBS, 10 mm (4‐(2‐hydroxyethyl)‐1‐piperazineethanesulfonic acid) (HEPES), 100 U mL^−1^ penicillin, 100 μg mL^−1^ streptomycin and 2 mm glutamine at 37°C for 15 min. Nonadherent cells were removed by replacing the culture medium. KCs were cultured for 6 h *in vitro*. Cells and supernatants were collected for further experiments.

### Western blot analysis

Proteins were extracted from liver tissue or cells with ice‐cold lysis buffer (50 mm Tris, 150 mm NaCl, 0.1% sodium dodecyl sulfate, 1% sodium deoxycholate, 1% Triton X‐100). Proteins (20 μg/sample) were subjected to 10% sodium dodecyl sulfate‐polyacrylamide gel electrophoresis and transferred to polyvinylidene difluoride nitrocellulose membranes (Bio‐Rad, Hercules, CA, USA). Monoclonal antirabbit NLRP3, cleaved caspase‐1, procaspase‐1, ASC, IL‐1β, pro‐IL‐1β, phospho‐AMPK, AMPK, phospho‐mTOR, mTOR, p62, LC3B, Bcl‐2, Bcl‐xL and β‐actin antibodies (Cell Signaling Technology) were used.

### Quantitative real‐time‐PCR

Total RNA was purified from cells with TRIzol reagent (Invitrogen, Carlsbad, CA, USA) according to the manufacturer's instructions. Reverse transcription into cDNA was performed with a Transcriptor First‐Strand cDNA Synthesis kit (Roche, Indianapolis, IN, USA). Quantitative real‐time PCR was performed using SYBR green (Roche) on a StepOnePlus Real‐Time PCR System (Applied Biosystems, Foster City, CA, USA). PCR amplification conditions were as follows: 95°C (30 s); 40 cycles of 95°C (5 s) and 60°C (30 s); and dissociation at 95°C (15 s), 60°C (60 s) and 95°C (15 s). Quantitative real‐time‐PCRs were all repeated three times. The expression levels of target genes and the results were normalized to Hypoxanthine phosphoribosyltransferase expression. The primer sequences used are shown in Supplementary table 1.

### Transmission electron microscopy

Transmission electron microscopy of KCs was performed according to the manufacturer's instructions. In brief, cells were treated with 1% OsO4, dehydrated with ethanol, embedded in Epon and then fixed with 2.5% glutaraldehyde at 4°C overnight. The embedded materials were then sectioned. The sections were further stained with 0.3% lead citrate and imaged with an electron microscope (HITACHI, Tokyo, Japan; 1200× or 5000× magnification).

### ELISA

IL‐1β and IL‐18 levels in sera or cell culture supernatants were measured using an ELISA kit (eBiosciences, San Diego, CA, USA) according to the manufacturer's protocols.

### Statistical analysis

Data were expressed as the mean ± s.e.m. Two group comparisons were performed using *t*‐test. Multiple group comparisons were performed using one‐way ANOVA followed by Bonferroni's *post hoc* test and were used for the analysis of multiple group comparisons. All statistical analyses were performed using STAT software, version 11.0. *P <* 0.05 (two‐tailed) was considered statistically significant.

## Conflict of Interest

All authors declare no conflict of interest.
