# Supplementary material for: Hyperglycemia aggravates acute liver injury by promoting liver‐resident macrophage NLRP3 inflammasome activation via the inhibition of AMPK/mTOR‐mediated autophagy induction
Source: Immunol Cell Biol. 2019 Nov 19;98(1):54–66. doi: 10.1111/imcb.12297 (PMC7004066; doi:10.1111/imcb.12297)
Supplement: Supplementary file 1 [file IMCB-98-54-s001.pdf]

Supplementary figure 1

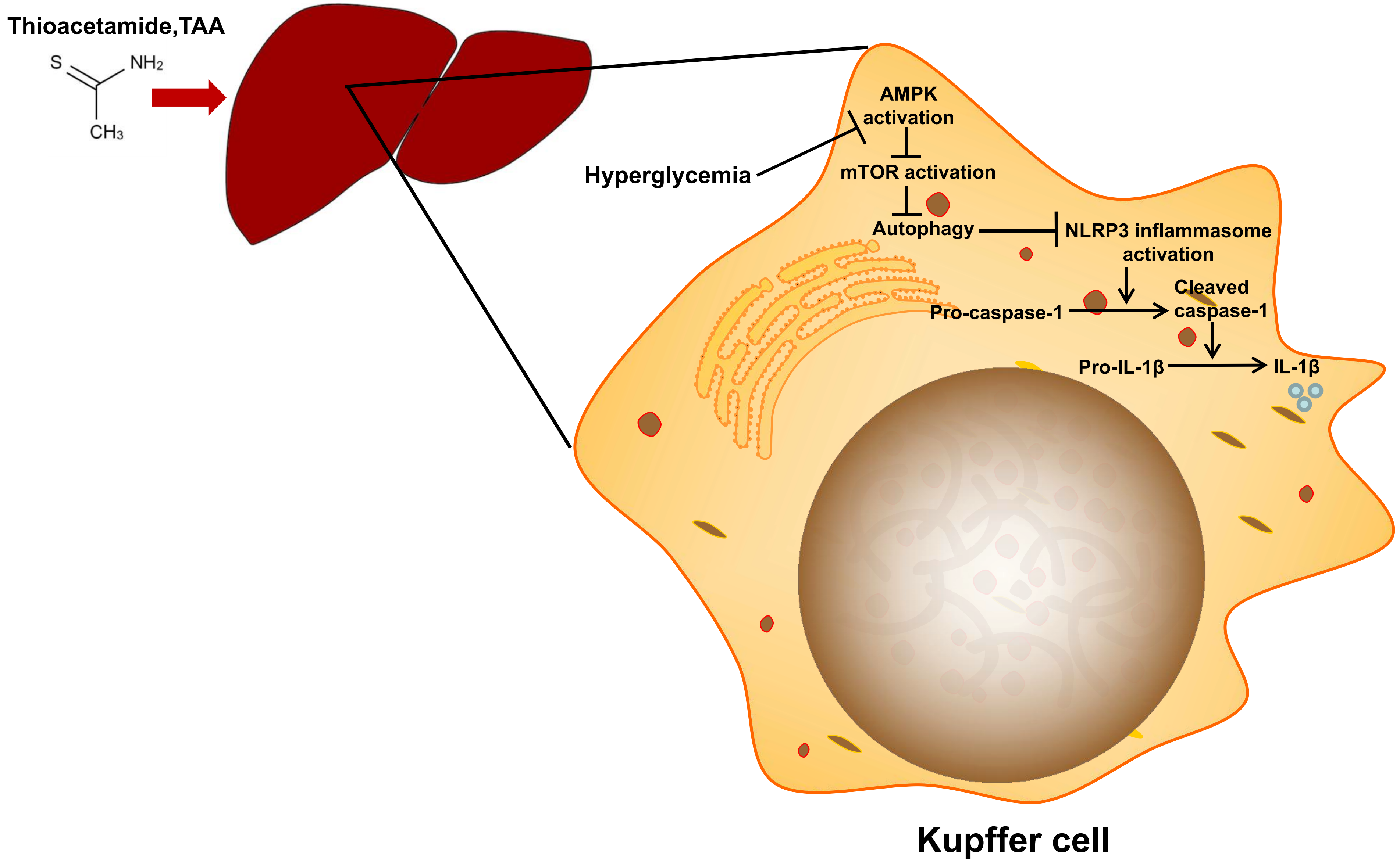

Supplementary figure 1 We demonstrated that hyperglycemia aggravated TAA-induced acute liver injury by promoting liver-resident macrophage NLRP3 inflammasome activation via inhibiting AMPK/mTOR-mediated autophagy. This study provides a novel target for the prevention of toxin-induced acute liver injury under hyperglycemia.
