# Supplementary material for: Hyperglycemia aggravates acute liver injury by promoting liver‐resident macrophage NLRP3 inflammasome activation via the inhibition of AMPK/mTOR‐mediated autophagy induction
Source: Immunol Cell Biol. 2019 Nov 19;98(1):54–66. doi: 10.1111/imcb.12297 (PMC7004066; doi:10.1111/imcb.12297)
Supplement: Supplementary file 2 [file IMCB-98-54-s002.pdf]

Supplementary table 1: Primer sequences for the amplification.

Gene	Forward Primer (5′ → 3′)	Reverse Primer (5′ → 3′)
NLRP3	5’-ATTACCCGCCCGAGAAAGG-3’	5’-TCGCAGCAAAGATCCACACAG-3’
IL-1β	5’-GCAACTGTTCCTGAACTCAACT-3’	5’-ATCTTTTGGGGTCCGTCAACT-3’
HPRT	5’-TCAGTCAACGGGGGACATAAA-3’	5’-GGGGCTGTACTGCTTAACCAG-3’
